# Three-dimensional mapping of cortical bone thickness in subjects with different vertical facial dimensions

**DOI:** 10.1186/s40510-016-0145-x

**Published:** 2016-10-17

**Authors:** Mais Medhat Sadek, Noha Ezat Sabet, Islam Tarek Hassan

**Affiliations:** Department of Orthodontics, Ain Shams University, Cairo, Egypt

**Keywords:** CBCT, Mini-implants, Cortical thickness, Facial type

## Abstract

**Background:**

The purpose of this study was to determine differences in cortical bone thickness among subjects with different vertical facial dimensions using cone beam computed tomography (CBCT).

**Methods:**

From 114 pre-treatment CBCT scans, 48 scans were selected to be included in the study. CBCT-synthesized lateral cephalograms were used to categorize subjects into three groups based on their vertical skeletal pattern. Cortical bone thickness (CBT) at two vertical levels (4 and 7 mm) from the alveolar crest were measured in the entire tooth-bearing region in the maxilla and mandible.

**Results:**

Significant group differences were detected with high-angle subjects having significantly narrower inter-radicular CBT at some sites as compared to average- and low-angle subjects.

**Conclusions:**

Inter-radicular cortical bone is thinner in high-angle than in average- or low-angle subjects in few selected sites at the vertical height in which mini-implants are commonly inserted for orthodontic anchorage.

## Background

The morphology of the craniofacial region is dominantly controlled by genetic factors. However, functional demands can have a significant effect on craniofacial growth and development [1]. Facial divergence has been related to the masticatory muscles, and the association between the hyperdivergent growth pattern and muscular hypofunction has previously been reported [2]. Changes in loading exerted by the muscles during function alter cortical bone thickness, not only at the site of muscle insertion but also in the alveolar bone of the tooth-bearing region of the jaws [3]. Accordingly, thickness of the cortical bone can provide an insight to the forces it experiences and is expected to vary in subjects with different vertical facial dimensions [4]. This was reported previously in which a significant, but complex relationship was found between cortical bone thickness and the facial type [5].

Recently, mini-implants have been extensively used as a reliable source of anchorage in orthodontics. Their small size, which allowed placement in inter-radicular areas; easy placement and removal; excellent anchorage; and low cost are main advantages [6]. Firm osseous support is the single most important factor for the success of an orthodontic mini-implant [7]. Cortical bone thickness (CBT) strongly affects biomechanical parameters of mini-implant bone interactions such as insertion torque and stress distribution [8, 9]. Recent studies were conducted to determine the optimal site for mini-implant placement inter-dentally based on measurements of cortical bone thickness using three-dimensional imaging [10–12] and skulls [13].

Furthermore, almost all studies that investigated the effects of CBT on the clinical success of mini-implants agreed that thin cortical bone is a real risk factor [9, 14, 15]. Motoyoshi et al. reported that CBT 1 mm or less is a risk factor for mini-implant failure [9]. Alrbata et al. found that the appropriate range of CBT for supporting an orthodontic mini-implant was from 1 to 2 mm [7].

Only two studies reported the relationship between facial divergence and cortical bone thickness measured inter-dentally at the vertical height in which mini-implants are commonly inserted for skeletal anchorage [16, 17]. Cortical bone was significantly thinner in high-angle patients when compared with low-angle patients, thereby posing increased risk of mini-implant failure in this group of patients. However, these two studies only studied the posterior region of the jaws despite the fact that mini-implants can be placed anteriorly for overbite correction as well as for space closure.

The purpose of this study was to investigate whether there are statistically significant differences in cortical bone thickness in the tooth-bearing region of the jaws, in the anterior as well as the posterior region, among subjects with different vertical facial dimensions, using cone beam computed tomography. This would provide reference data for clinicians placing mini-implants in subjects with different facial types.

## Methods

Cone beam computed tomography scans of 114 subjects, aged between 18 and 35 years old, were analyzed. Those scans were collected from the Department of Oral and Maxillofacial surgery and were taken for selected cases as a part of pre-extraction assessment of impacted mandibular third molars. For some of these cases, a large field of view was taken where imaging for the upper third molars was also needed, and these were the scans included in the study. These tomographs were obtained by iCAT cone beam computed tomography (CBCT) scanner (Model 17/19 series; Imaging Sciences International, Hatfield, Pennsylvania, USA) at the following settings: 120 kVp at 5 mA for a total scan time of 7 s, with a voxel size 0.3 mm.

Subjects receiving previous or current orthodontic treatment, obvious periodontal disease (determined from radiographic signs of bone loss), missing permanent teeth (excluding third molars), severely ectopic teeth (such as buccally erupting canines), and evidence of previous trauma were excluded. The remaining 48 scans were then included in the study. Subjects’ rights were protected, and approval was obtained from the university research ethics committee.

**Fig. 1:**
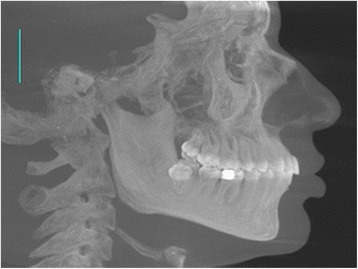
CBCT-synthesized lateral cephalometric radiograph

The three-dimensional image was reconstructed by iCATVision™ software (version 1.7.0.7, Imaging Sciences International) and saved in digital imaging and communications in medicine (DICOM) format. Subjects were classified into three groups based on their facial type as determined from lateral cephalograms synthesized from the CBCT scans using the maximum intensity projection technique (Fig. 1). The CBCT-synthesized lateral cephalogram was then saved as JPEG image and imported into Onyx ceph™ software (version 2.6.52, Image Instruments, Chemnitz, Germany).

Facial type categories were determined using the following cephalometric measurements: (1) facial height index (the ratio of posterior facial height to anterior facial height): it is equal to 66.2 ± 3.3 % in patients with an average growth pattern [18] and (2) mandibular plane angle (the angle between the anterior cranial base (sella to nasion, SN) and the mandibular plane (formed from menton to gonion, Me-Go): it is equal to 32.5° ± 3.4° in patients with an average growth pattern [18]). Patients had to fit into a single category for both measurements to be included in the study. Three subjects who fell into mixed categories on a single cephalogram were excluded from the groups. Subjects were divided according to facial type: 17 with an average vertical facial dimension—normal-angle group (10 women, 7 men), 13 with a high vertical facial dimension—high angle group (7 women, 6 men), and 15 with a low vertical facial dimension—low angle group (8 women, 7 men).

**Fig. 2:**
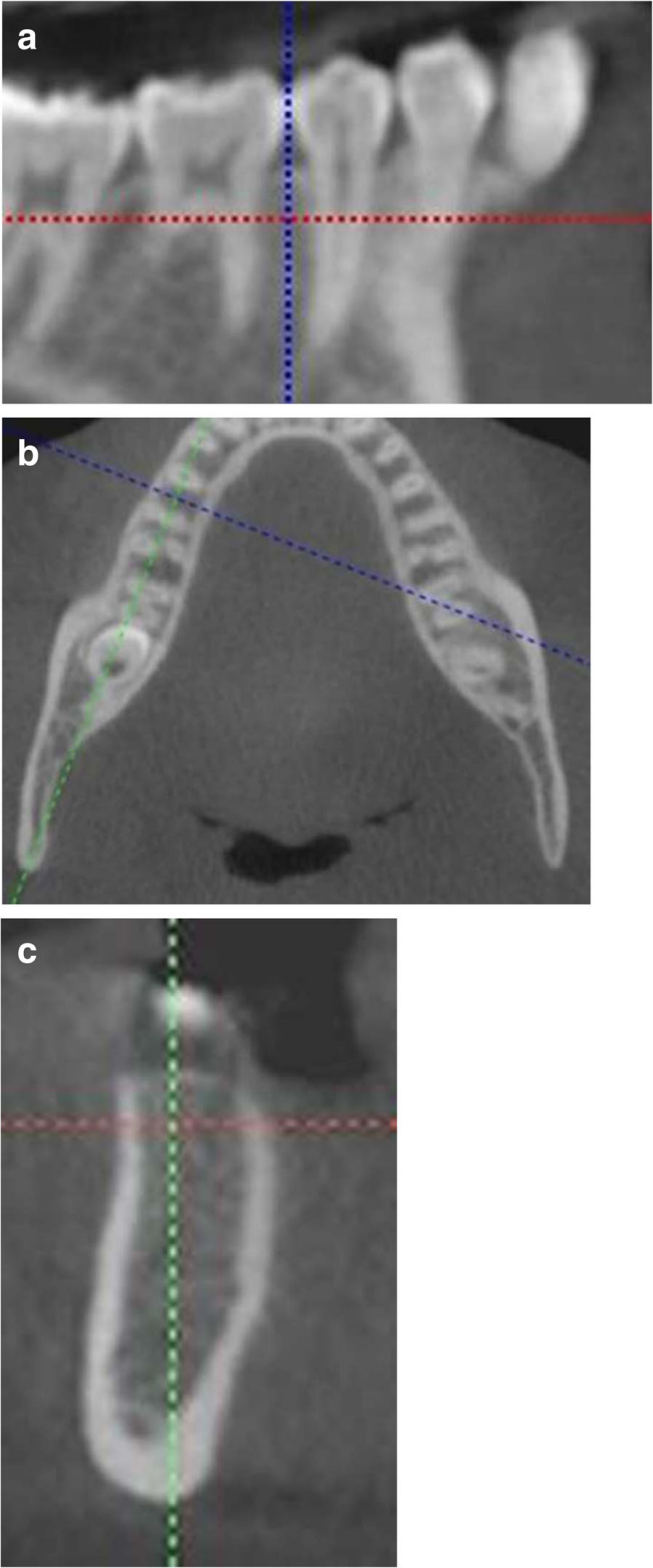
Orientation of views for the inter-radicular measurements. **a** Orientation of sagittal slice. **b** Orientation of axial slice. **c** Orientation of coronal slice

Using iCATVision™ software, two-dimensional slices, 0.3-mm thick, through each contact area were created. Orientation of each site in all three planes of space was carried out before measurement. The inter-radicular area of interest was located on the sagittal slice (Fig. 2a). The slice was then oriented so that the inter-radicular space was bisected by the vertical reference line and was parallel to the long axes of the roots. Orientation of the axial slice was then used to ensure that the horizontal reference line bisected the inter-radicular area and traversed the thinnest area of cortical bone (Fig. 2b). The horizontal reference line was moved to establish the measurement level in relation to the alveolar crest as seen on the coronal slice (Fig. 2c) [10].

**Fig. 3:**
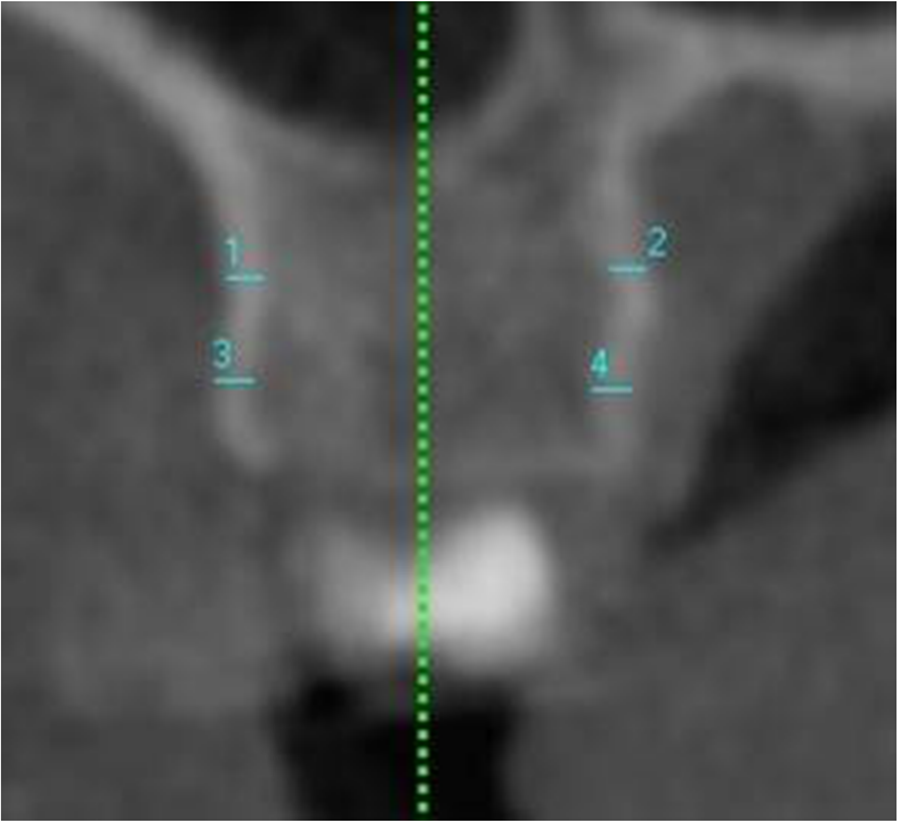
Coronal section through the inter-radicular area between upper right first and second molars. Buccal cortical plate thickness at 4 mm (measurement *3*) and at 7 mm (measurement *1*) apical to the crest of the alveolar bone. Palatal cortical plate thickness at 4 mm (measurement *4*) and at 7 mm (measurement *2*) apical to the crest of the alveolar bone
- On the palatal side of the maxillary teeth, palatal cortical bone thickness was measured at 4 and 7 mm apical to the crest of the alveolar bone. The lingual cortical plate in the mandible was not measured because of its limited use for mini-implant placement [13].

For each inter-radicular space in the maxilla and mandible, from the second molar on one side to the second molar on the opposite side, the following measurements were done:

- Labial/buccal CBT at 4 and 7 mm apical to the crest of the alveolar bone. It was defined as the thickness of the labial/buccal cortical plate measured perpendicular to the bone surface (Fig. 3).

To reduce fluctuations in measurement accuracy in this study, one trained orthodontist made all measurements. The intra-operator error was obtained by repeating measurements by the same observer, 2 weeks apart, on ten randomly selected subjects. Inter-operator error measurements were evaluated by having other trained orthodontic operator take measurements on the same subjects. The intra-operator and inter-operator error was assessed using the intra-class correlation coefficient. High correlation was found for both intra-operator (*r* = 0.998) and inter-operator (*r* = 0.997) error.

Numerical data were explored for normality by checking the data distribution, calculating the mean and median values, evaluating histograms and normality curves, and using Kolmogorov–Smirnov and Shapiro–Wilk tests. Data were presented by mean and standard deviation. Kruskal–Wallis test with statistical significance considered at a *P* level lower than 0.05. When significantly different, further pair-wise comparisons were done with the Mann–Whitney *U* tests with the Bonferroni adjustment. Statistical analysis was performed with IBM SPSS Statistics Version 20 for Windows (IBM Corporation, NY, USA. SPSS, Inc., an IBM Company).

## Results

In the upper arch, on the buccal side, comparison of inter-radicular CBT measurements among the three groups revealed statistically significant differences at few selected sites primarily located mesial and distal to the first molar at the 4-mm level (Table 1) and mesial to the first molar at the 7-mm level (Table 2). Palatally, no statistically significant differences were found at any site at the 4-mm level between the three groups (Table 3), while statistically significant differences were found mesial and distal to the lateral incisor at the 7-mm level (Table 4). The low-angle group showed the thickest cortical plate while the high-angle group showed the thinnest.

**Table 1:** Measurements of buccal inter-radicular cortical bone thickness at 4 mm from the alveolar crest in the upper arch

		Mean	Std. deviation	*P* value
CBT at midline	High angle	.8333	.17386	0.874
	Normal	.8450	.10464	
	Low angle	.8786	.05669	
CBT at 1/2	High angle	.8750	.02739	0.685
	Normal	.9017	.19156	
	Low angle	.8814	.41894	
CBT at 2/3	High angle	.9333	.13292	0.545
	Normal	.9400	.15556	
	Low angle	.9886	.20772	
CBT at 3/4	High angle	.8950	.17479	0.253
	Normal	.9867	.15358	
	Low angle	.9886	.13108	
CBT at 4/5	High angle	.9383	.07223	0.888
	Normal	.9717	.14077	
	Low angle	.9757	.12232	
CBT at 5/6	High angle	.9000 a	.03162	0.003*
	Normal	.9357 ab	.12952	
	Low angle	1.0883 b	.02440	
CBT at 6/7	High angle	.9167 a	.02582	0.035*
	Normal	.9633 ab	.06055	
	Low angle	1.1457 b	.21953	

Descriptive statistics, overall significance by Kruskal–Wallis test, and the results of pair-wise comparisons with the Mann–Whitney *U* test with the Bonferroni adjustment

*Significant at *P* ≤ 0.05. Different letters are statistically significantly different

**Table 2:** Measurements of buccal inter-radicular cortical bone thickness at 7 mm from the alveolar crest in the upper arch

		Mean	Std. deviation	*P* value
CBT at midline	High angle	.9033	.17049	0.862
	Normal	.9000	.06325	
	Low angle	.9200	.14877	
CBT at 1/2	High angle	.9083	.03764	0.306
	Normal	.9600	.10789	
	Low angle	.9257	.43428	
CBT at 2/3	High angle	.9883	.12497	0.298
	Normal	1.0083	.20024	
	Low angle	1.1286	.16046	
CBT at 3/4	High angle	.9250	.02739	0.114
	Normal	1.1000	.24916	
	Low angle	1.1057	.14421	
CBT at 4/5	High angle	.9667	.11690	0.66
	Normal	1.0583	.15651	
	Low angle	1.0614	.22638	
CBT at 5/6	High angle	.9000 a	.03162	0.046*
	Normal	.9383 ab	.15092	
	Low angle	1.0614 b	.15614	
CBT at 6/7	High angle	1.0517	.16005	0.613
	Normal	1.0833	.12144	
	Low angle	1.1471	.20862	

Descriptive statistics, overall significance by Kruskal–Wallis test, and the results of pair-wise comparisons with the Mann–Whitney *U* test with the Bonferroni adjustment

*Significant at *P* ≤ 0.05. Different letters are statistically significantly different

**Table 3:** Measurements of palatal inter-radicular cortical bone thickness at 4 mm from the alveolar crest in the upper arch

		Mean	Std. deviation	*P* value
CBT at 1/2	High angle	.8833	.04082	0.092
	Normal	1.0800	.14241	
	Low angle	.9271	.55087	
CBT at 2/3	High angle	.6083	.47267	0.084
	Normal	1.0417	.17058	
	Low angle	1.0771	.62152	
CBT at 3/4	High angle	.99	.257	0.114
	Normal	1.25	.095	
	Low angle	1.21	.298	
CBT at 4/5	High angle	.8083	.41282	0.432
	Normal	1.0650	.31002	
	Low angle	1.0171	.15185	
CBT at 5/6	High angle	.9683	.21470	0.624
	Normal	1.0050	.11292	
	Low angle	.9800	.07071	
CBT at 6/7	High angle	1.10	.180	0.557
	Normal	.93	.026	
	Low angle	1.01	.144	

Descriptive statistics, overall significance by Kruskal–Wallis test, and the results of pair-wise comparisons with the Mann–Whitney *U* test with the Bonferroni adjustment

*Significant at *P* ≤ 0.05

**Table 4:** Measurements of palatal inter-radicular cortical bone thickness at 7 mm from the alveolar crest in the upper arch

		Mean	Std. Deviation	*P* value
CBT at 1/2	High angle	.9217	.08612	0.032*
	Normal	1.2350 a	.23990	
	Low angle	1.1157 a	.54851	
CBT at 2/3	High angle	.7183	.56201	0.045*
	Normal	1.213 a	.10838	
	Low angle	1.1614 a	.56304	
CBT at 3/4	High angle	1.25	.399	0.435
	Normal	1.35	.118	
	Low angle	1.30	.188	
CBT at 4/5	High angle	.8633	.49017	0.125
	Normal	1.2333	.21379	
	Low angle	1.2286	.16067	
CBT at 5/6	High angle	.8750	.48587	0.41
	Normal	1.1517	.25733	
	Low angle	1.1429	.15239	
CBT at 6/7	High angle	1.10	.180	0.836
	Normal	.98	.129	
	Low angle	1.06	.229	

Descriptive statistics, overall significance by Kruskal–Wallis test, and the results of pair-wise comparisons with the Mann–Whitney *U* test with the Bonferroni adjustment

*Significant at *P* ≤ 0.05. Different letters are statistically significantly different

On the other hand, in the lower arch, statistically significant differences were found in the mandibular posterior region, mesial and distal to the first molar at the 4-mm level (Table 5) and at the region between first and second premolars, as well as mesial and distal to the first molar at the 7-mm level (Table 6). Similar to the upper arch, the low-angle group showed the thickest cortical plate while the high-angle group showed the thinnest.

**Table 5:** Measurements of buccal inter-radicular cortical bone thickness at 4 mm from the alveolar crest in the lower arch

		Mean	Std. deviation	*P* value
CBT at midline	High angle	0.91	0.18	0.726
	Normal	0.83	0.11	
	Low angle	0.91	0.14	
CBT at 1/2	High angle	0.95	0.11	0.800
	Normal	0.87	0.20	
	Low angle	0.92	0.11	
CBT at 2/3	High angle	0.90	0.14	0.572
	Normal	1.01	0.14	
	Low angle	1.02	0.15	
CBT at 3/4	High angle	0.96	0.16	0.461
	Normal	1.01	0.16	
	Low angle	1.02	0.09	
CBT at 4/5	High angle	1.01	0.22	0.082
	Normal	1.27	0.20	
	Low angle	1.37	0.14	
CBT at 5/6	High angle	1.09 c	0.19	0.005*
	Normal	1.48 b	0.14	
	Low angle	1.71 a	0.11	
CBT at 6/7	High angle	1.51 b	0.14	0.029*
	Normal	1.75 a	0.13	
	Low angle	1.85 a	0.20	

Descriptive statistics, overall significance by Kruskal–Wallis test, and the results of pair-wise comparisons with the Mann–Whitney *U* test with the Bonferroni adjustment

*Significant at *P* ≤ 0.05. Different letters are statistically significantly different

**Table 6:** Measurements of buccal inter-radicular cortical bone thickness at 7 mm from the alveolar crest in the lower arch

		Mean	Std. deviation	*P* value
CBT at midline	High angle	0.91	0.08	0.428
	Normal	1.01	0.08	
	Low angle	0.92	0.11	
CBT at 1/2	High angle	1.00	0.10	0.905
	Normal	1.01	0.10	
	Low angle	1.02	0.07	
CBT at 2/3	High angle	0.95	0.09	0.055
	Normal	1.24	0.07	
	Low angle	1.11	0.10	
CBT at 3/4	High angle	1.04	0.10	0.061
	Normal	1.19	0.11	
	Low angle	1.26	0.07	
CBT at 4/5	High angle	1.16	0.12	0.007*
	Normal	1.33	0.21	
	Low angle	1.56	0.18	
CBT at 5/6	High angle	1.31 c	0.08	<0.001*
	Normal	1.65 b	0.18	
	Low angle	1.85 a	0.20	
CBT at 6/7	High angle	1.96 b	0.21	<0.001*
	Normal	2.05 b	0.17	
	Low angle	2.33 a	0.09	

Descriptive statistics, overall significance by Kruskal–Wallis test, and the results of pair-wise comparisons with the Mann–Whitney *U* test with the Bonferroni adjustment

*Significant at *P* ≤ 0.05. Different letters are statistically significantly different

## Discussion

This study compared cortical bone thickness among subjects with different vertical facial dimensions in the entire tooth-bearing region of both jaws, using CBCT. This aimed to provide reference data for clinicians that will aid in mini-implant placement in subjects with varying facial types.

Cortical bone thickness is the key determinant of initial stability of mini-implants, and thin cortical bone was reported to increase the risk of mini-implants failure [19]. On the other hand, areas with thick cortical bone can increase the risk of mini-implant breakage and bone micro-fractures. This can pose two important questions: Do subjects with different vertical facial dimensions have different cortical bone thickness? And if yes, what are the clinical implications of such differences on mini-implant stability?

Masumoto et al. through measurements of cortical bone thickness on 31 dry skulls of modern Japanese males found that cortical bone thickness of the mandibular first and second molar sections was thicker in short-faced subjects than in average- and long-faced subjects [5]. Swasty et al. investigated differences in CBT in patients with different vertical facial dimensions using CBCT [20]. It was reported that the long-faced group had thinner cortical bone in almost all sites. Unfortunately, this study measured CBT at one third and two thirds the distance from the base of the mandible to the alveolar crest, rather than using a standard site for the measurements.

Similar to our study, Ozdemir et al. measured CBT at 4 mm from the alveolar crest, which appears to correspond to the attached gingiva [17]. This was reported to be a favorable area for mini-implant placement, considering the lower probability of inflammation. They found a close relation between facial type and cortical bone thickness, at the inter-dental sites from the distal aspect of the canine to the mesial aspect of the second molar. Cortical bone thickness in the low-angle group was significantly higher than in the high-angle group in all four measured sites.

In our study, significant group differences were detected with high-angle subjects having significantly narrower inter-radicular CBT at some sites as compared to average- and low-angle subjects. These were in the posterior region of the maxilla and mandible on the buccal side and palatally in the maxilla mesial and distal to the lateral incisor, at the vertical height in which mini-implants are commonly inserted for orthodontic anchorage.

Horner et al. reported that the cortical bone was 0.08 to 0.64 mm thicker in the hypodivergent than in the hyperdivergent subjects [16]. This is similar to the findings of our study in which the cortical bone was 0.16 to 0.62 mm thicker in the hypodivergent than in the hyperdivergent subjects.

Furthermore, the results of this study may be correlated with the findings of previous studies [20, 21], in which a significant relationship was found between facial type and alveolar thickness. High-angle subjects were found to have thinner alveolus at almost all sites in the mandible. This could be associated with the finding that these are the same subjects that showed a thinner cortical plate in the posterior region of the mandible when compared to normal- or low-angle cases.

Such differences in CBT among subjects with different vertical facial dimensions can have significant clinical implications. An association between higher risk of failure of mini-implants and subjects with high mandibular plane angle has been previously reported [15]. On the other hand, Kuroda et al. insisted that there was no correlation between the success rate of mini-implants and the mandibular plane angle [22]. From the results of our study, it appears that although there is a correlation between the vertical facial dimensions and inter-radicular cortical bone thickness at the vertical height in which mini-implants are commonly inserted, this is evident in only few sites: primarily located in the posterior region of the maxilla and mandible on the buccal side and palatally in the anterior region of the maxilla. More studies are thus needed to determine the exact relationship between the vertical skeletal pattern and the success rate of mini-implants.

Another clinically related important fact is that high-angle subjects when compared to the other two groups tended to have more sites with cortical bone thickness less than 1 mm, which according to Motoyoshi et al. can increase the risk of failure of mini-implants placed at these sites [9].

This should merit our attention to take precautions to increase the success rate of mini-implants placed in high-angle patients, especially if they are an important part of our treatment plan. This can include to monitor and emphasize oral hygiene more strictly or to use partially osseointegrated mini-implants that may offer higher stability. Another option would be to use mini-plates which were reported to be associated with a lower failure rate than mini-implants [23]. Furthermore, extensive research is needed exploring different ways to promote mini-implant stability as they are being used more and more in our practice.

Perhaps most importantly, the results give clinicians reference data for measurements of cortical bone thickness for subjects with different vertical skeletal patterns. However, several factors must be taken into consideration when evaluating the results. *First*, this study did not investigate the difference between male and female subjects. However, in a recent study by Farnsworth et al., no sex differences in cortical thickness in either the maxilla or the mandible were found between males and females [10]. Since maximum bite force is not a regular or habitual function, like mastication, for example, it might not be expected to produce sex differences in cortical thickness. *Second*, inherent limitations of CBCT imaging should also be considered [24]. Partial volume averaging can influence the spatial resolution. Thin bone is especially susceptible to partial volume averaging. *Third*, the differences in the density of cortical bone were not evaluated in this study. In future research, we should also evaluate bone mineral density of cortical bone. As well as bone quantity (bone thickness), bone quality (mineralization) can affect initial stability values for orthodontic mini-implants [6].

## Conclusions

Inter-radicular cortical bone was thinner in high-angle subjects, compared to the low- and normal-angle groups, in the posterior region of the maxilla and mandible on the buccal side as well as palatally in the maxilla mesial and distal to the lateral incisor, at the vertical height in which mini-implants are commonly inserted for skeletal anchorage. High-angle subjects tended to have more sites with cortical bone thickness less than 1 mm.

## Abbreviations

CBCT: Cone beam computed tomography
CBT: Cortical bone thickness
